# The 2018 National Report on Children’s and Adolescents’ Physical Activity and Physical Fitness for Montenegro

**DOI:** 10.18502/ijph.v49i10.4705

**Published:** 2020-10

**Authors:** Bojan MASANOVIC, Stevo POPOVIC, Dusko BJELICA, Jovan GARDASEVIC

**Affiliations:** 1. Faculty for Sport and Physical Education, University of Montenegro, Niksic, Montenegro; 2. Montenegrin Sports Academy, Podgorica, Montenegro

**Keywords:** Children, Adolescents, Physical activity, Physical fitness, Monitoring, Montenegro

## Abstract

**Background:**

We aimed to focus on a review of the literature on physical activity and physical fitness for children and adolescents in Montenegro, to identify and evaluate the current situation and provide a better basis for the creation of a future monitoring system.

**Methods:**

The Montenegrin Research Team (MRT) has set up a specific methodology that is reflected in the assessment of physical activity and physical fitness of Montenegrin children and adolescents through ten separate indicators. Grades were awarded based on data found in available scientific articles that were published up to 2018, as well as in the grey literature, such as government and nongovernment reports and online content from the same period.

**Results:**

All indicators averaged 3.7 on a six-point scale. It is interesting to note that two indicators were rated as excellent, three more indicators were rated with above-average, four indicators were rated as below-average and, lastly, one indicator was rated as “unfinished”.

**Conclusion:**

Children’s and adolescents’ physical activity and physical fitness for Montenegro might be good, especially if compared to other countries from the region.

## Introduction

Physical inactivity and increasing screen-time among children and adolescents around the world have been well-documented in the previous three decades (1) and they are two of the leading causes of poor physical fitness among the members of those populations. Likewise, several recognized methodologies actively monitor the physical activity and physical fitness of children and adolescents worldwide (2–4), however, they are locally oriented, and when it comes to Montenegro, this vital issue has remained unexplored.

Led by experience from neighbouring countries, Montenegro has recognized the need to establish a system for monitoring and promoting physical activity and physical fitness in children and adolescents. In the process of creating a new system, based on the experience of one of the best European examples, SLOfit, the Slovenian national surveillance system for physical and motor development of children and young people (5), there was a need to collect all available data at the national level and make a systematic review that will help to create the system that can satisfy the specific needs of requires Montenegrin society.

Therefore, we aimed to focus on a review of the literature on physical activity and physical fitness for children and adolescents in Montenegro, to identify and score the current situation and provide a better basis for the creation of a future monitoring system. An additional objective is to identify narrower areas of research that are currently lacking or underdeveloped in Montenegro and find the best way to solve issues regarding the current physical inactivity and increasing screen-time and improve opportunities for children and adolescents across the country to be much more active and outside of closed areas.

## Methods

The Montenegrin Research Team (MRT), established by Montenegrin Sports Academy, has set up a specific methodology according to the previous good practices from neighbouring countries and beyond. This methodology is reflected in the assessment of physical activity and physical fitness of Montenegrin children and adolescents (aged 6–19 yr) through ten separate indicators (total physical activity, active sports, unstructured active play, active transportation, sedentary behaviours, physical fitness, parental support, activity at school, safe environment, governmental promotion and control). Furthermore, to evaluate each of these indicators, a separate systematic analysis was conducted, and a six-point grading scale was employed (6=excellent; 5=very good; 4=good; 3=fair; 2=poor; 1=without reliable information). Grades were defined when the percentage of children meeting established benchmarks fell below a defined level: 6 = 81% to 100%; 5 = 61% to 80%; 4 = 41% to 60%; 3 = 21% to 40%; 2 = 0% to 20%; 1 = without reliable information. The grades were awarded based on data found in available scientific articles that were published up to 2018 (with no restriction on the earliest date), as well as in the grey literature, such as government and nongovernment reports and online content from a specific period. The electronic databases (Google Scholar and Web of Science) were searched for research articles, the website of the Ministry of Sports were searched for government and nongovernment reports and online content needed to evaluate the tenth indicator. When a conclusion had to be drawn from multiple studies, the grade was a weighted average between the groups. The data were synthesized, and a set of benchmarks was used to assign the grades. Two researchers (members of the MRT) were rate the studies, if there were any deviations the final grades were completed upon consensus.

## Results

Grades for each indicator were awarded based on different numbers and quality of studies. The ratings for each indicator are shown in Table 1.

**Table 1:** The 2018 National Report Grades

***Indicator***	***Grade***	***Rationale***
Total physical activity	**2**	34.71% of children accumulate at least 20 minutes self-selected physical activity and running 4 days or more per week, according to information reported by their parents (6).
Active sports	**3**	25% of children and youth participate in organized sport or physical activities programmes, according to information reported by their parents (6).
Unstructured active play	**4**	44.98% of children play actively at least 30 minutes per day, according to information reported by their parents (6).
Active transportation	**1**	There is no study that contains data on how many children and youth in Montenegro use active transportation to get to and from places.
Sedentary behaviours	**6**	94.2% of parents report that their children spend no more than 2 hours of screen-time on an average day (6).
Physical fitness	**4**	The average percentile achieved in physical fitness was 58.75% in boys and 55.63% in girls based on the international normative values (8).
Parental support	**6**	87.26% participate in physical activities with children, and 30.03% are provide material support and paid a fee to ensure that children can attend sports classes, according to information reported by parents (6).
Activity at school	**5**	According to official data from Ministry of Education 100% schools provide mandatory physical education hours 2 to 3 time per week to their students, while a majority of students are taught by a physical education specialist in 45% elementary schools and 100% secondary schools (9, 10, 11).
Safe environment	**3**	55.01% of the parents indicated that their neighbourhood environment was safe to some extent and safe for their child to play outside with other children from the neighbourhood, without adult supervision (6).
Ministry promotion and control	**2**	Ministry promotion strategies and initiatives is more geared towards professional sports less to physical activity for children, while progress through the policy evaluation are also unknown (based on reports available on the website of the Ministry of Sports (12).

### Total physical activity

Only one study used objective tools to evaluate the overall physical activity of children and adolescents in Montenegro (6). The sample included 1356 parents of pre-school children from each Montenegrin city, while the results showed that 34.71% of children accumulate at least 20 min of self-selected physical activity and running four days or more per week. In addition, 49.7% of children accumulate at least 20 min of self-selected physical activity and running three days or more per week, while 15.5% of children accumulate at least 20 min of self-selected physical activity and running two or fewer days per week. Although data on the number of active children exist, it is not possible to estimate with certainty how many of them meet the minimum recommended by the W H O, which is 60 min of moderate to vigorous physical activity per day (7). Therefore, the overall physical activity indicator was graded as poor (grade 2), a degree lower than it would be by the percentages.

### Active sports

Based on the study mentioned in the previous paragraph, an evaluation of the participation of children and adolescents in organized sport was performed (6).

The results show that only 25% of children participate in organized sport or physical activity programmes. Most of them were involved in the practice of basketball clubs (28.41%), dance clubs (22.52%), football clubs (19.30%), martial arts clubs (17.42%), 4.02% tennis clubs, while other children (8.33%) were found their place in other sports clubs. The active sports participation indicator was graded as fair (grade 3).

### Unstructured active play

For the evaluation of the participation of children and adolescents in unstructured active play, unfortunately, the study of Bjelica et al (6), was again the only existing source. According to the results from that study, 44.98% of children play actively at least 30 min per day, and 64.53% of children prefer to play outdoors than in their homes. From all of the above, the unstructured active play indicator was graded as good (grade 4).

### Active transportation

A study containing information on how many children and adolescents in Montenegro use active transportation to get to and from places does not exist. It is interesting to note that there is a general opinion that cities in Montenegro do not have enough bikeways and that traffic is not safe enough for cyclists (13). The low use of walking or bicycling could be related to the lack of pedestrian and bicycle infrastructure, as well as the actual or perceived dangers of walking and cycling (14). Of course, this does not necessarily mean that the number of children walking or cycling to school is related to the development level of a country (14). The active transportation indicator was graded without reliable information (grade 1) and should be explored in future studies.

### Sedentary behaviors

Data on combined screen-time (television, computer, gaming, internet, mobile phone) during the day was also assessed (6). This study indicates that 94.2% of children meet the recommendation of fewer than two hours of screen-time per each day. Sixty five percent of parents reported that their children spend between one and two hours watching television and videos or playing games on the computer or the video (at home, in kindergarten, at a neighbour’s) on an average day, while 29.87% of parents reported that their children’s spend less than one hours of screen-time on an average day. This fact showed that children in Montenegro have excellent screen-related habits. Therefore, the grade for this indicator was excellent (grade 6).

### Physical fitness

Physical fitness plays a key role in a child’s healthy growth and development, which Montenegrin experts recognized and researched. Physical fitness evaluation was based on the results of eight research studies conducted in Montenegro up to 2018. The sample included a total of 808 boys and 399 girls, 13 to 16-yr-old schoolchildren from different Montenegrin cities (15–22). In each of these studies, the EUROFIT test battery was applied to measure components of physical fitness, which made this analysis sufficiently compatible. The average percentile achieved in fitness capacity was 58.75% in boys and 55.63% in girls. The percentiles achieved by Montenegrin boys and girls were compared with the international normative values of physical fitness (8), based on which the physical fitness indicator was graded as good (grade 4).

### Parental support

Family is one of the most important influences on participation in sports and physical activity; however, research on this topic is limited. 87.26% of parents participated in physical activities with children, 95.43% of parents encouraged children to be physically active, 75.05% were providing logistic support for physical activity, 30.03% provided material support and paid a fee to ensure that children can attend sports classes (6). This fact confirms that a high percentage of parents took part in children’s physical activity and encouraged them to engage in some activities by themselves; consequently, the parental support in Montenegro was graded as excellent (grade 6).

### Activity at school

In Montenegro, physical education is a compulsory subject in all primary and secondary schools. According to official data from the Ministry of Education (9, 10), 100% of schools in Montenegro provide mandatory physical education hours two to three times per week to their students. The number of required physical education classes vary by grade. From 1st to 6th grade, pupils attend three compulsory physical education classes per week, while from 7th to 9th grade pupils attend two compulsory classes per week. In all secondary school grades, students attend two compulsory physical education classes per week. The percentage of physical education classes taught by a physical education specialist also varies by grade; from 1st to 3rd grade, physical education is taught by a classroom teacher; in 4th and 5th grades, 50% classes can be taught equally classroom teacher and specialists; and from 6th grade onward through secondary school, 100% of physical education classes are taught by physical education specialists with a university degree (11). The majority of students are taught by a physical education specialist in 45% of elementary schools and 100% of secondary schools.

Finally, regarding school sports infrastructure, in Montenegro over 60% of the schools have halls for physical education, while those schools that do not have outdoor courts where physical education classes can be organized (23). Moreover, it is important to note the following: a very low percentage of students (lower than 0.5%), do not attend physical education classes for various reasons (24). The activity at school was overall graded as very good (grade 5).

### Safe environment

Only one study provides some information about how safe the environment is in Montenegro (6). The results of this repeatedly mentioned study show that 50% of the parents indicated that their neighbourhood environment is safe to some extent for their child to play outside with other children from the neighbourhood, while 5.01% of the parents indicated that their neighbourhood environment is safe for their child to play outside with other children from the area without adult supervision. Unfortunately, this study does not provide data on how parents consider the safety of their children because it would help resolve the problem of security and give some specific recommendations (6). This study also confirmed the fact that the parents do not allow their children to play outside if there is fear regarding security.

In contrast, parents have a firm attitude the if the security issue might be ameliorated, participation in physical activity would surely increase, which might improve several indicators from this study, and enable them to have a higher rating. Considering that most of those parents who think their environment is safe, however, make that claim that the environment is currently somewhat safe, this category was graded as fair (grade 3), a degree lower than it would be if expressed as a percentage.

### Governmental promotion and control

According to official data from Ministry of Sports (12) and reports that are located on the site (25), it is clear that promotion strategies and initiatives exist. However, they are more focused towards professional sports less to physical activity for children, while progress through the policy evaluation is unknown. Given the lack of multi-annual strategy that would improve physical activities opportunities for children and adolescents across the country, ministry promotion and control was graded at 2 (poor).

## Discussion

It is essential to discuss the fact that this is the first national report study that monitors children’s and adolescents’ physical activity and physical fitness from Montenegro in accordance to some standardized methodology. There have been several previous initiatives, but this is the first system-based study set up on the methodologically correct approach that is recognized in the neighbouring and more distant countries across Europe and throughout the world. Physical inactivity and increasing screen-time among children and adolescents around the world have been well-documented in the previous three decades, but the Montenegrin case has not been studied yet; this is the first time the scientific audience will have access to this kind of systematic review. According to the results synthesized from the available data, all indicators in this study averaged 3.7 (slightly closer to good than fair) on a six-point scale. It is interesting to highlight that two indicators were rated as excellent: sedentary behaviours and parental support. This fact confirms that children and adolescents in Montenegro have excellent screen-related habits, better than their counterparts in France, Germany, Chile, and Nigeria (26, 27, 28, 29), and a high percentage of parents take part in children’s and adolescents’ physical activity and encourage them to take some activities by themselves, which is in accordance with the habits of their counterparts in Germany and Canada (27, 30).

In contrast, three more indicators were rated with above-average marks. Activity at school is rated with a very good mark, and the number of mandatory lessons and the professional qualifications of teachers is in parallel to those of children and adolescents from the Czech Republic (31), while the number of mandatory lessons and the professional qualifications of teachers is still worse than those of students in Slovenia (32). Unstructured active play and physical fitness are marked as good, which is in accordance with the habits of children and adolescents from the Czech Republic (31), while the habits of children and adolescents from Ethiopia and Brazil are somewhat better (33, 34). These facts indicated that activity at school was confirmed through very good scores in three areas: number of physical education classes, a high percentage of qualified personnel, and a low number of students who do not attend physical education classes for various reasons. Furthermore, it is very important to highlight there is a high percentage of children who daily participate in the unstructured play, while children and adolescents achieved good scores on certain physical fitness indicators based on the EUROFIT normative values.

In contrast to the previously mentioned, four indicators were rated as fair and poor: active sports, safe environment, total physical activity, ministry promotion, and control. The facts from this part of the study indicated that only one-quarter of subjects were active in the sports clubs and that almost half of the parents think that the children are not safe when they are playing in the neighbourhood. That is in parallel with results for the same indicators in Chile (28), but rather worse than the results in Lithuania and Germany (14, 27).

Also, total physical activity among children and adolescents in Montenegro is not enough, mostly because that only one-third of the subjects were at least 20 min active in extracurricular physical activity, for a minimum of four days a week, and that ministry promotion strategies and initiatives are geared more towards professional sports and less to physical activity for children and multi-annual strategies that can improve physical activities opportunities for children and adolescents. These indicators are rated much worse than in countries such as Canada and Slovenia (30, 32), and ranked among Chile and Ethiopia (28, 33).

Lastly, it must not be forgotten that one indicator was rated as unfinished (active transportation), which means there was no active transportation research contained in the searched databases in this study.

This study is essential as it represents a cornerstone in terms of creating a system for monitoring the physical activity and physical fitness of children and adolescents in Montenegro. It is also vital for the practitioners and policymakers, mostly because it identified the current situation in Montenegro though reviewing all available scientific articles as well as grey literature, such as government and nongovernment reports and online content, and can be used an initial step in planning an adequate development strategy at the national level. Based on this study, policy-makers will be able to use the summarized results and offer the practitioners the best opportunities to improve the physical activity and physical fitness for children and adolescents in Montenegro. Lastly, scientists will recognize the missing areas and launch research that can cover overlooked areas and improve the general knowledge in this field study.

Children’s and adolescents’ physical activity and physical fitness for Montenegro might be good, especially if compared to other countries from the region (Slovenia, Croatia, Serbia, Bosnia and Herzegovina, Kosovo, Albania, etc.). The active lifestyle of children and adolescents in Montenegro should continue to be intensively promoted because the volume of total physical activity and the number of children actively involved in sport must be increased. Efforts should be directed towards improving the safety of the environment as much as possible, because the fact that only 50% of parents feel that their children are safe in the environment is alarming. If this is accomplished, and children and adolescents have more diverse content in the environment, the enjoyment of unstructured active play can also be improved. Also, the level of activity of children and adolescents in school should be increased, which would certainly lead to physical fitness achieving the highest values. Promising foundations, which suggest that the situation may be much better in the future, are the excellent support provided by parents to their children, also the fact that Montenegrin children and adolescents do not have the habit of sitting in front of the computer more than they should.

Although this study has so many benefits, and its presence is more than welcome, there are many limitations, so it is difficult to defend all the conclusions previously specified. If we review the quantity and the quality of the studies based on which the grades were awarded, it is poor and leaves some serious gaps in our research. Namely, out of ten indicators, six were evaluated based on the results of only one study (6), mostly because there were no other studies investigating these issues in Montenegrin society. Although significantly used, the previously mentioned study focused only on pre-school children, so any conclusion that included the entire population of children and adolescents might have serious issues. Furthermore, some indicators were not evaluated according to the primary sources, and the secondary sources, such as reports authored by unqualified staff, might cause significant damage if they are provided as credible facts. It is also important to highlight that some limitations are reflected in the fact that some data sources for physical fitness indicators have serious methodological limitations (18, 20–22).

In addition to all the above and unmentioned limitations, this study is of great value because it combined in one study all that is available in the areas of physical activity and physical fitness in children and adolescents in Montenegro. Also, the authors of this study appeal to all those who can help to enrich a wider database and establish a precise system for monitoring the physical activity and physical fitness of children and adolescents in Montenegro to do so, and invite any kind of research that would cover already existing variables, but especially those which were not covered in this study, as this would be beneficial for the significant improvement of the conclusions and recommendations in the next National Report on Children’s and Adolescents’ Physical Activity and Physical Fitness for Montenegro, which is expected in 2020.

## Ethical considerations

Ethical issues (Including plagiarism, informed consent, misconduct, data fabrication and/or falsification, double publication and/or submission, redundancy, etc.) have been completely observed by the authors.
